# Electrical activity and fatigue of respiratory and locomotor muscles in obstructive respiratory diseases during field walking test

**DOI:** 10.1371/journal.pone.0266365

**Published:** 2022-04-01

**Authors:** Jéssica D. Cavalcanti, Guilherme Augusto F. Fregonezi, Antonio J. Sarmento, Thiago Bezerra, Lucien P. Gualdi, Francesca Pennati, Andrea Aliverti, Vanessa R. Resqueti

**Affiliations:** 1 Departamento de Fisioterapia, Laboratório PneumoCardioVascular—Hospital Universitário Onofre Lopes / Empresa Brasileira de Serviços Hospitalares & Laboratório de Inovação Tecnológica em Reabilitação, Universidade Federal do Rio Grande do Norte, Natal, Rio Grande do Norte, Brasil; 2 Faculdade de Ciências da Saúde do Trairí, Universidade Federal do Rio Grande do Norte, Santa Cruz, Rio Grande do Norte, Brasil; 3 Dipartimento di Elettronica, Informazione e Bioingegneria, Politecnico di Milano, Milan, Italy; Universita degli Studi di Milano, ITALY

## Abstract

**Introduction:**

In subjects with obstructive respiratory diseases the increased work of breathing during exercise can trigger greater recruitment and fatigue of respiratory muscles. Associated with these changes, lower limb muscle dysfunctions, further contribute to exercise limitations. We aimed to assess electrical activity and fatigue of two respiratory and one locomotor muscle during Incremental Shuttle Walking Test (ISWT) in individuals with obstructive respiratory diseases and compare with healthy.

**Methods:**

This is a case-control study. Seventeen individuals with asthma (asthma group) and fifteen with chronic obstructive pulmonary disease (COPD group) were matched with healthy individuals (asthma and COPD control groups). Surface electromyographic (sEMG) activity of sternocleidomastoid (SCM), scalene (ESC), and rectus femoris (RF) were recorded during ISWT. sEMG activity was analyzed in time and frequency domains at baseline and during the test (33%, 66%, and 100% of ISWT total time) to obtain, respectively, signal amplitude and power spectrum density (EMG median frequency [MF], high- and low-frequency bands, and high/low [H/L] ratio).

**Results:**

Asthma group walked a shorter distance than controls (p = 0.0007). sEMG amplitudes of SCM, ESC, and RF of asthma and COPD groups were higher at 33% and 66% of ISWT compared with controls groups (all p<0.05). SCM and ESC of COPD group remained higher until 100% of the test. MF of ESC and RF decreased in asthma group (p = 0.016 and p < 0.0001, respectively) *versus* controls, whereas MF of SCM (p < 0.0001) decreased in COPD group compared with controls. H/L ratio of RF decreased (p = 0.002) in COPD group *versus* controls.

**Conclusion:**

Reduced performance is accompanied by increased electromyographic activity of SCM and ESC and activation of RF in individuals with obstructive respiratory diseases during ISWT. These are susceptible to be more pronounced respiratory and peripheral muscle fatigue than healthy subjects during exercise.

## Introduction

Obstructive respiratory diseases, such as asthma and chronic obstructive pulmonary disease (COPD), are characterized by persistent respiratory symptoms, including dyspnea, wheeze, coughing, and increased sputum production associated with limited expiratory airflow [1, 2]. During exercise, ventilatory demand increases and overloads inspiratory muscles [3, 4]. with disproportionate increase in neural respiratory drive directed towards the respiratory muscles [5, 6]. Increased work of breathing during exercise may lead to respiratory muscle fatigue and worsen the sensation of dyspnea. Individuals with asthma and COPD can present dysfunction of locomotor muscles [7–11] due to changes in peripheral structures and energy metabolism [7, 12], increasing the risk of peripheral fatigue during exercise.

Muscle fatigue is defined as a reduction in maximum capacity to generate force or power output [13] resulted from insufficient oxygen, supply of nutritive substances, and decreased removal of local by-products [14]. In this context, the lower blood flow disponibility may increase susceptibility to early peripheral fatigue since the increased work of breathing on exercise, deviating blood flow from locomotor to respiratory muscles [15, 16]. Therefore, events associated with intrinsic changes in locomotor muscles may impact exercise tolerance in individuals with obstructive respiratory diseases [17].

Surface electromyography (sEMG) is a valuable technique to assess muscle function [18] and provide real-time information about muscle activation and fatigue development [19]. sEMG signal amplitude is sensitive to motor unit recruitment and represents the magnitude of muscle activation [20], whereas changes in power spectrum density are attributed to muscle fiber conduction velocity [21] and activity of remaining slow motor units [14]. Changes in power spectrum can also indicate fatigue development through methods, such as median frequency (MF), high- and low-frequency bands, and high/low (H/L) ratio [22, 23].

Mechanical abnormalities in individuals with obstructive respiratory diseases alter the load-capacity balance of the respiratory system, contributing to greater neural respiratory drive and inspiratory muscle recruitment during exercise. Also, locomotor muscles dysfunction associated with blood flow competition between respiratory and locomotor muscles may further compromise symptoms, leading to early exercise interruption. Cardiopulmonary exercise test is commonly used to identify exercise limitation due to respiratory and locomotor muscle commitments [24]; however, activation of these muscles during field walking tests that reproduce daily activities (e.g., incremental shuttle walking test [ISWT]) has been under-investigated. We hypothesize that individuals with chronic respiratory diseases have greater activations and develop respiratory and locomotor muscles fatigue during daily activities, compared to healthy individuals. Therefore, this study aimed to assess respiratory and locomotor muscles activation and fatigue development during ISWT in individuals with obstructive respiratory diseases.

## Methods

This case-control study followed the Declaration of Helsinki and was approved by the research ethics committee of the Hospital Universitário Onofre Lopes (HUOL)/Empresa Brasileira de Serviços Hospitalares (EBSERH) (number 1.344.501). All participants signed the informed consent form.

### Subjects

**Fig 1:**
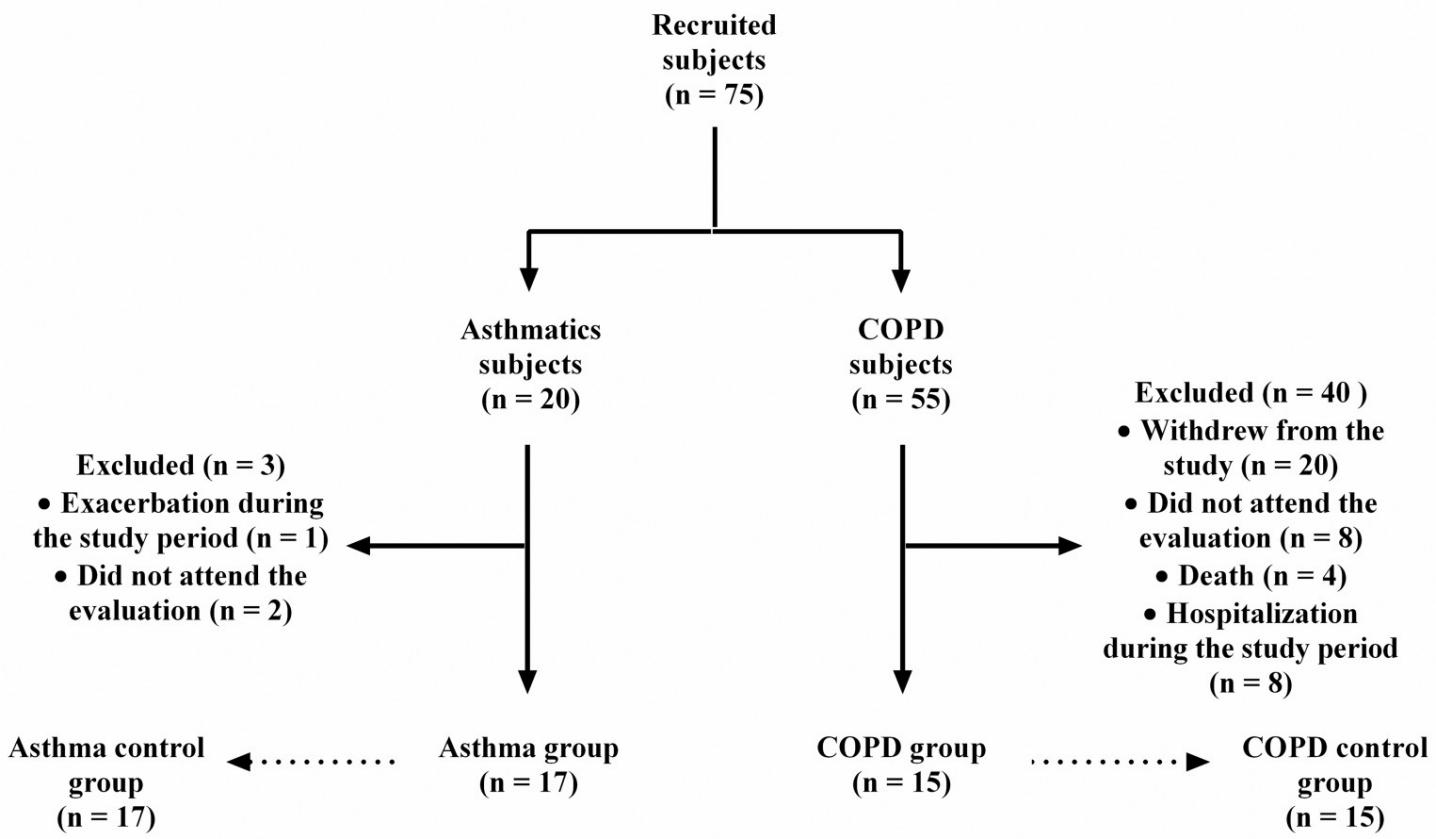
Flowchart selection of participants.

Were included 17 Individuals with clinical diagnosis of asthma (asthma group) (aged between 25–60 years) and 15 COPD subjects (COPD group) (aged between 50–80 years), from both genders, under medical supervision, without disease exacerbation in the last four weeks, body mass index less than 30 kg/m^2^, and non-smokers. The difference in age range between groups was due to clinical phenotype of late-onset asthma that starts in adulthood [1]. For COPD subjects, age was chosen due to highest prevalence among those aged >60 years in Brazil [2]. All individuals were matched for age, sex, and BMI with self-reported healthy individuals, non-smokers, and without impaired lung function (17 matched with asthmatics [asthma control group] and 15 matched with COPD subjects [COPD control group]) (Fig 1). Individuals with asthma-COPD overlap syndrome, cognitive or osteoarticular deficits compromising data collection, and those who exacerbated during the study period were excluded.

### Study design

The study protocol was conducted in two moments: 1) anthropometric data (shown in Table 1), pulmonary function and respiratory muscle strength assessment and 2) ISWT. Assessments were performed in two non-consecutive days with maximum interval of seven days in between.

**Table 1:** Anthropometric characteristics of the sample.

	*Asthma group (n = 17)*	*95%CI*	*Control group (n = 17)*	*95%CI*	*Difference (95%CI)*	*p*	*COPD group (n = 15)*	*95%CI*	*Control group (n = 15)*	*95%CI*	*Difference (95%CI)*	*p*
Age (years)	34.76 ± 11.18	29.01–40.51	33.88 ± 12.66	27.37–40.39	-0.88 (-9.22–7.46)	0.83	65.6 ± 7.84	61.26–69.94	65.13 ± 7.79	60.82–69.45	-0.46 (-6.31–5.38)	0.87
Sex	F: 15 M: 2	-	F: 15 M: 2	-	-	-	F: 10 M: 5	-	F: 10 M: 5	-	-	-
Body weight (kg)	62.84 ± 9.19	58.11–67.57	61 ± 5.11	58.37–63.63	-1.84 (-7.03–3.35)	0.47	71.93 ± 12.23	65.16–78.70	64.53 ± 13.79	56.89–72.17	-7.4 (-17.15–2.34)	0.13
Height (m)	1.60 ± 0.10	1.54–1.65	1.64 ± 0.07	1.60–1.68	0.03 (-0.02–0.10)	0.22	1.57 ± 0.06	1.53–1.60	1.54 ± 0.11	1.48–1.61	-0.02 (-0.09–0.04)	0.44
BMI (Kg/m^2^)	24.66 ± 4.08	22.56–26.76	22.65 ± 2.02	21.6–23.69	-2.01 (-4.26–0.24)	0.07	28.97 ± 3.91	26.80–31.14	26.84 ± 3.29	25.01–28.66	-2.13 (-4.84–0.57)	0.11

Data reported as mean ± standard deviation. F: female. M: male. Kg: kilograms. m: meters. BMI: body mass index. Kg/m^2^: kilogram per square meter.

### Pulmonary function and respiratory muscle strength

Pulmonary function was assessed by spirometry test using the Koko® spirometer (model 313106, Longmont, USA), following reproducibility and acceptability criteria of the American Thoracic Society/European Respiratory Society [25]. Post-bronchodilator spirometry was performed in individuals with asthma and COPD using 400 mcg of salbutamol sulfate. Reversibility criteria followed GINA [1] recommendations for individuals with asthma and GOLD [2] recommendations for individuals with COPD. Absolute values and percentage of predicted forced vital capacity (FVC), forced expiratory volume in the first second (FEV_1_) and FEV_1_/FVC ratio values were considered and compared with predicted for the Brazilian population [26].

Respiratory muscle strength was assessed by volitional measurement of maximal respiratory pressure at the mouth (Maximal Inspiratory Pressure—MIP and Maximal Expiratory Pressure–MEP) and at nostril (Sniff Nasal Inspiratory Pressure–SNIP). We used a digital manovacuometer (NEPEB—LabCare/ UFMG, Belo Horizonte—MG, Brazil) to measure MIP, MEP, and SNIP tests following the American Thoracic Society/European Respiratory Society Statement [18]. Predicted values for MIP and MEP followed Neder et al. [27], while Araújo et al. [28] were used for SNIP.

### Incremental shuttle walking test

The ISWT was used to assess the exercise capacity, according to Singh et al. [29]. The primary result is the distance walked in meters. Subjects walked in a flat corridor at a 10 meters distance around two cones placed 9 meters apart. The two cones were inset 0.5 m from either end to avoid the need for abrupt changes in direction. The walking speed was determined by an audio signal, in which the test level changed every minute, stimulating the participant to increase their walking speed. Predicted walking distances were calculated according to Jürgensen et al. [30]. Respiratory rate (RR), dyspnea, and lower limb fatigue (modified Borg scale 0–10) were evaluated before and after ISWT. The RR was evaluated by counting respiratory incursions in one minute. Heart rate and peripheral oxygen saturation (SpO_2_) were monitored before, during, and after the test using a pulse oximeter (MODEL 2500ª, Plymouth, Minnesota—USA).

### sEMG activity

sEMG activity of sternocleidomastoid (SCM), scalene (ESC), and rectus femoris (RF) muscles were recorded during ISWT. A TeleMyo DTS Desk Receiver electromyograph (Noraxon®, Scottsdale- USA) with four wireless sensors (Noraxon®, Scottsdale—USA), 16-bit resolution, and common-mode rejection ratio of >100 dB was used. sEMG data were captured at a sampling frequency of 1500 Hz, with low-pass filter of 500 Hz, amplified 1000 times, and stored in the MR 3.12 software (Noraxon®, Scottsdale—USA). Bipolar electrodes with 20 mm inter-electrode distance and contact surface in gel and silver/silver chloride were used.

Skin preparation followed SENIAM guidelines (Surface EMG for Non-Invasive Assessment of Muscles) [31]. Electrode positioning followed anatomical references and muscle fiber orientation of SCM (lower third of the distance between sternoclavicular joint and mastoid process) [32], ESC (five centimeters from sternoclavicular joint and two centimeters above this point) [33], and RF (half distance between upper anterior iliac spine and superior portion of the patella) [34]. All electrodes were positioned on the right side of the body to avoid noise and interference from cardiac electrical activity.

### sEMG signal processing

Time-domain analysis was performed to obtain signal amplitudes (i.e., root mean square [RMS]) at baseline, 33%, 66%, and 100% of ISWT total time using MR 3.12 software (Noraxon, Scottsdale-USA). Raw sEMG data were filtered with a high-pass filter, cut-off frequency at 20 Hz, rectified with full-wave rectifier, and smoothened with RMS algorithm in a time window of 50 ms. Electrocardiogram filter was applied to reduce noise caused by cardiac activity. RMS data were normalized by peak value achieved during ISWT [35].

Frequency domain analysis was performed to identify fatigue development as a shift of the sEMG power spectrum toward lower frequencies [36]. sEMG signals were processed offline using MATLAB R2018a (Mathworks, Natick, MA). Power spectral density was calculated applying the continuous Wavelet transform technique using Daubechies 4 mother wavelet. For each power spectrum, the median frequency was calculated. Analysis was performed in 5-s time windows, and sEMG signals were digitized with a band-pass filter (fourth-order Butterworth filter, 20–500 Hz).

For a complete analysis of changes in power spectrum density, relative contributions of high- and low-frequency bands and H/L frequency ratio were computed by filtering the signal with different bandpass filters and integrating each filtered output to obtain high- and low- frequency powers. Shifts in spectral properties of frequency bands (e.g., lower overall power spectrum) are linked to changes in motor unit recruitment priority (e.g., higher contribution of low-frequency motor units) [37]. For respiratory muscles, high-frequency band was defined at 130–250 Hz and low frequency at 30–50 Hz [18]. For peripheral muscles, high and low-frequency bands were defined as 194–250 Hz and 27–48 Hz, respectively [37]. Frequency bands and H/L ratio were normalized for each participant and expressed as percentage of values obtained in the initial 10 s of the test. This normalization allowed comparison between participants since fatigue was not present at the beginning of the test.

Since MF and H/L ratio may show different decay patterns during the task, one of the following criteria should be met to confirm fatigue development: 1) negative slopes for linear regressions, representing displacement of frequency to lower values [38] or 2) decrease below 60% of values recorded at the beginning of the test for exponential regressions, as observed in human diaphragm and skeletal muscles [39].

### Sample size calculation and statistical analysis

Sample size was calculated in a pilot study with asthma and healthy subjects (GPower 3.1 software) using RMS of SCM at 100% total ISWT time. With an effect size of 0.8, 80% statistical power, and significance level of 0.5, a sample size of 21 participants was estimated. GraphPad Prism 6.0 software was used to perform statistical analyses, assuming a significance level of p < 0.05. Shapiro-Wilk test verified data normality. Unpaired t-tests were performed to analyze anthropometric data, lung function, and ISWT performance. Mann-Whitney test was performed for cardiorespiratory variables, dyspnea, lower limb fatigue, and sEMG activity. Friedman with Dunn’s post-hoc was performed for intragroup analyzes regarding ISWT moments and baseline. Regression analysis was also performed to determine r^2^ and extract slopes for MF, high- and low-frequency bands, and H/L ratio during ISWT. Slopes of regression were also calculated between groups by the F-test. For Mann-Whitney test, Cohen’s d was calculated to estimate effect sizes, and values interpreted as large (0.5), medium (0.3), and small (0.1) [40]. For Friedman test, effect sizes were estimated using Kendall’s W coefficient, ranging from 0 (no relationship) to 1 (perfect relationship) [41].

## Results

The final sample of individuals with obstructive respiratory diseases was classified as six individuals from the asthma group with mild (35%) and 11 (64%) with moderate asthma disease [1]. Eight individuals from the COPD group had GOLD 2 (53%) and seven (46%) as GOLD 3 [2]. Pulmonary function and respiratory muscle strength data of the sample studied are shown in Table 2.

**Table 2:** Pulmonary function data of the sample.

	*Asthma group (n = 17)*	*95%CI*	*Control group (n = 17)*	*95%CI*	*Difference (95%CI)*	*p*	*COPD group (n = 15)*	*95%CI*	*Control group (n = 15)*	*95%CI*	*Difference (95%CI)*	*p*
FVC (L)	3.37 ± 0.72	3–3.75	3.51 ± 0.57	3.21–3.89	0.13 (-0.32–0.59)	0.55	1.88 ± 0.44	1.64–2.13	2.82 ± 0.47	2.56–3.09	0.93 (0.59–1.28)	<0.0001*
FVC(%pred)	91.41 ± 13.60	84.42–98.40	92.03 ± 8.6	87.57–96.50	0.62 (-7.35–8.59)	0.87	63.46 ± 13.73	55.85–71.06	98.12 ± 19.40	87.38–108.9	34.66 (22.09–47.23)	<0.0001*
FEV_1_ (L)	2.36 ± 0.57	2.07–2.66	2.87 ± 0.54	2.59–3.15	0.50 (0.11–0.90)	0.01*	1.17 ± 0.30	1–1.34	2.18 ± 0.36	1.98–2.39	1.01 (0.76–1.26)	<0.0001*
FEV_1_(%pred)	77.20 ± 17.23	68.34–86.06	91.16 ± 6.25	87.95–94.38	13.96 (4.90–23.02)	0.003*	48.72 ± 15.81	39.96–57.48	95.24 ± 19.74	84.31–106.2	46.52 (33.14–59.89)	<0.0001*
FEV_1_/FVC	0.70 ± 0.10	0.64–0.75	0.82 ± 0.05	0.80–0.85	0.12 (0.06–0.18)	0.0001*	0.62 ± 0.07	0.58–0.67	0.77 ± 0.07	0.73–0.82	0.15 (0.09–0.21)	<0.0001*
MIP_%pred_	91.86 ± 23.34	79.86–103.9	113.9 ± 27.07	100–127	22.08 (4.42–39.74)	0.015*	89.21 ± 23.65	76.11–102.3	90.05 ± 19.79	79.10–101	0.84 (-15.46–17.16)	0.91
MEP_%pred_	86.22 ± 19.93	75.97–96.46	116.8 ± 26.98	102.9–130.7	30.58 (14.01–47.15)	0.0007*	92.85 ± 13.55	85.35–100.4	95.89 ± 16.98	86.49–105.3	3.04 (-8.45–14.53)	0.59
SNIP_%pred_	79.84 ± 23.37	67.83–91.85	98 ± 22	86.69–109.3	18.16 (2.30–34.02)	0.026*	70.94 ± 20.28	59.71–82.17	81.05 ± 10.96	74.98–87.12	10.11 (-2.07–22.30)	0.10

Data reported as mean ± standard deviation. FVC: forced vital capacity. FEV_1_: forced expiratory volume in the first second. FEV_1_/FVC: FEV_1_/FVC ratio. MIP: maximal inspiratory pressure. MEP: maximal expiratory pressure. SNIP: sniff nasal inspiratory pressure. L: liters %_pred_: % predicted.

*Unpaired t test.

### ISWT performance

Walking distance was 26% shorter for asthma group than asthma control group in both absolute (p = 0.007; Cohen’s d = 0.45) and predicted values (p = 0.0001, Cohen’s d = 0.45). No difference (p > 0.05) was found between COPD group and COPD control group. However, at the end of ISWT, COPD group showed lower SpO_2_ and greater RR, dyspnea, and lower limb fatigue than COPD control group (Table 3).

**Table 3:** Results of ISWT.

*ISWT*												
	*Asthma group (n = 17)*	*95%CI*	*Control group (n = 17)*	*95%CI*	*Difference (95%CI)*	*p*	*COPD group (n = 15)*	*95%CI*	*Control group (n = 15)*	*95%CI*	*Difference (95%CI)*	*p*
ISWT (m)	445.1± 126	380.3–509.8	607.8±127	542.5–673.1	162.8 (74.39–251.1)	0.0007*	275.3 ± 87.74	226.7–323.9	302.7 ± 91.53	252–353.4	27.33 (-39.73–94.39)	0.41
ISWT (%pred)	71.78±18.01	62.52–81.04	93.40±9.43	88.55–98.25	21.62 (11.57–31.66)	0.0001*	67.84 ± 19.35	57.12–78.55	73.63 ± 22.25	61.31–85.95	5.79 (-9.80–21.39)	0.45
Borg D _(0–10)_	3 [0.5–3]	1.14–3.26	3 [2–4]	1.86–3.31	0 (-1–2)	0.35	2 [1–3]	1.58–2.41	1 [0–1]	0.54–1.3	-1 (-2 - -1)	0.001^┼^
Borg F _(0–10)_	1[0.5–4]	1.18–3.40	2 [1–4]	1.37–3.21	1 (-1–1.5)	0.80	4 [2–5]	2.88–4.58	3 [2–3]	2.25–2.95	-1 (-2–0)	0.025^┼^
HR _(bpm)_	78 [73.5–92]	87.46–113.1	124 [101–143]	106.5–133.4	24 (2–42)	0.03^┼^	93 [87–100]	87.5–97.44	89 [81–101]	83.61–97.19	-4 (-12–6)	0.45
SpO_2 (%)_	98 [97–98.5]	96.72–98.57	98 [98–99]	97.65–98.70	0 (-1–1)	0.66	92 [90–94]	90.25–93.49	96 [96–97]	95.5–96.9	4 (3–6)	< 0.0001^┼^
RR _(Bpm)_	20 [18–22]	20.6–25.53	24 [20–29.5]	21.13–26.99	0 (-3–5)	0.67	21 [18–22]	19.3–21.9	18 [16–20]	17.26–19.14	-3 (-4 - -1)	0.004^┼^

Data presented as mean ± standard deviation for ISWT distance and ISWT%pred. Data presented as medians [interquartile 25%– 75%] for D (dyspnea), F (fatigue), HR (heart rate), SpO_2_ (oxygen saturation) and RR(respiratory rate). bpm: beats per minute. Bpm: breath per minute %pred: % predicted. m: meters.

* Unpaired t test.

^┼^ Mann-Whitney Test: p<0.05 between groups.

### Amplitude of EMG signals during ISWT

#### Sternocleidomastoid muscle (SCM)

**Fig 2:**
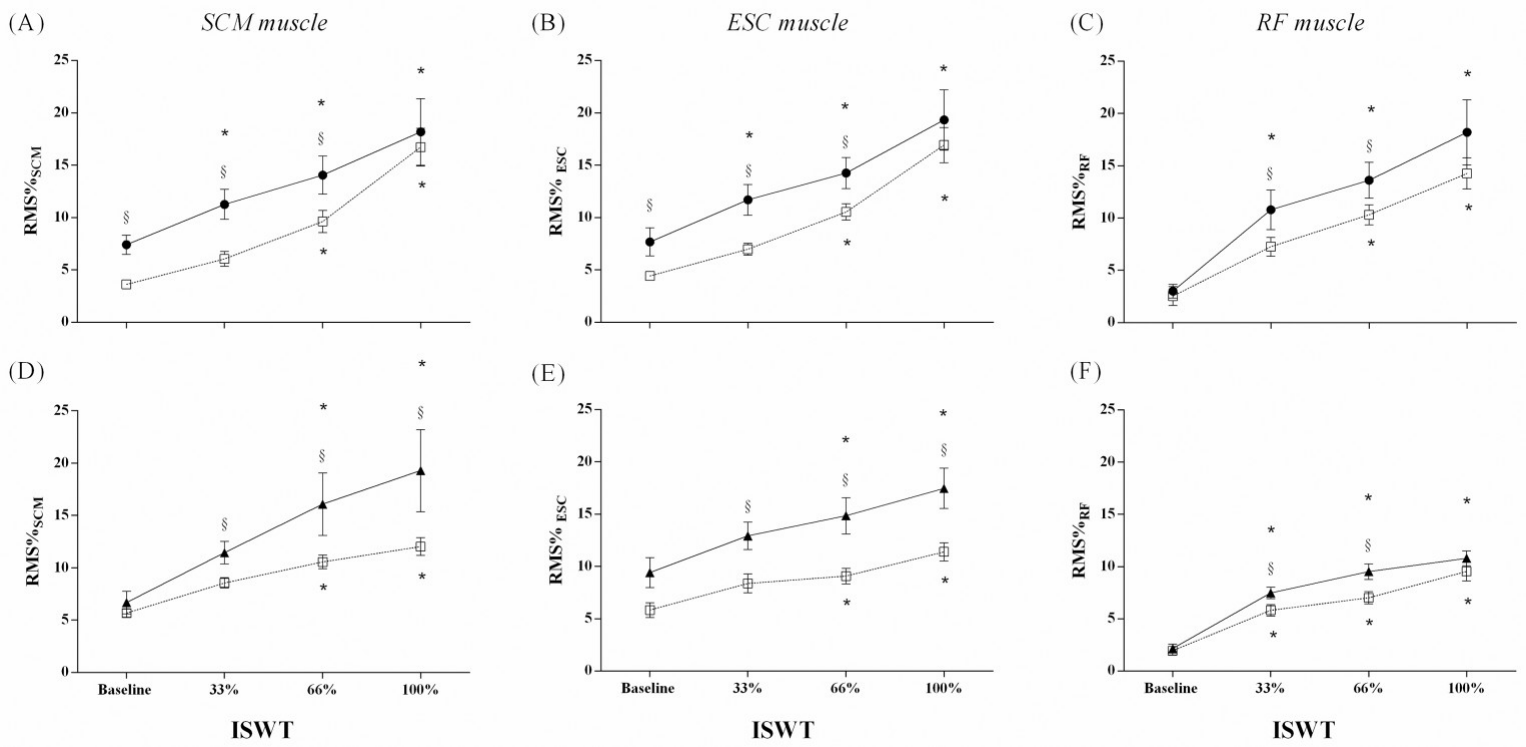
sEMG amplitude of SCM (A and D), ESC (B and E), and RF (C and F) muscles at baseline and during ISWT moments, in Asthma-group (close circles) on upper panel, COPD-group (close triangles) on the lower panel, and their respective control group (open square). ^§^ Mann-Whitney Test: difference between groups. *Friedman Test: compared to baseline.

%RMS of SCM was greater in asthma group than asthma control group at baseline (p = 0.003), 33% (p = 0.0005), and 66% (p = 0.004) of total ISWT time. %RMS of SCM in asthma group increased immediately after initiating the test (33% ISWT moment) and continued rising until the end of the test (p < 0.0001, Kendall’s W = 0.83). In contrast, sEMG activity of SCM increased in asthma control group only between 66% and 100% of total ISWT time (p < 0.0001, Kendall’s W = 0.88) (Fig 2A). %RMS of SCM was also greater in COPD group than COPD control group at 33% (p = 0.009), 66% (p = 0.023), and 100% (p = 0.023) of total ISWT time. %RMS of SCM increased between 66% and 100% of total ISWT time in both COPD (p < 0.0001, Kendall’s W = 0.70) and COPD control groups (p < 0.0001, Kendall’s W = 0.81) (Fig 2D).

#### Scalene muscle (ESC)

%RMS of ESC was greater in asthma group than asthma control group at baseline (p = 0.017), 33% (p = 0.001), and 66% (p = 0.033) of total ISWT time. In asthma group, %RMS of ESC increased almost immediately after initiating the test (p < 0.0001, Kendall’s W = 0.81), while in asthma control group the activity increased between 66% and 100% of total ISWT time (p < 0.0001, Kendall’s W = 0.86) (Fig 2B). %RMS of ESC was higher in COPD group than COPD control group at 33% (p = 0.006), 66% (p = 0.008), and 100% (p = 0.016) of total ISWT time. Similar to SCM, both groups demonstrated increased %RMS of ESC between 66% and 100% of total ISWT time (p < 0.0001, Kendall’s W = 0.69 for COPD group and p < 0.0001, Kendall’s W = 0.61, for COPD control group) (Fig 2E).

#### Rectus femoris (RF)

%RMS of RF was greater in asthma group at 33% (p = 0.02) and 66% (p = 0.004) of total ISWT time. In asthma group, %RMS of RF increased immediately after initiating ISWT (33% ISWT moment) (p < 0.0001, Kendall’s W = 0.82). In asthma control group, %RMS of RF increased from 66% of total ISWT time (p < 0.0001, Kendall’s W = 0.87) (Fig 2C). COPD group was significantly different from COPD control group at 33% (p = 0.032) and 66% (p = 0.039) of total ISWT time. In both groups, %RMS of RF also increased at 33% of ISWT (p < 0.0001, Kendall’s W = 0.86 for the COPD group and p < 0.0001, Kendall’s W = 0.82 for the COPD control group) (Fig 2F). Effect sizes and differences [95%CI] between groups for all muscles evaluated are shown in Table 4.

**Table 4:** Effect size and difference (95% CI) values of EMGs amplitude between groups.

	*Asthma group (n = 17) vs*. *Control group (n = 17)*			*COPD group (n = 15) vs*. *Control group (n = 15)*	
		Cohen’s *d*	*Difference (95%CI)*	Cohen’s *d*	*Difference (95%CI)*
SCM	Baseline	0.56	-3.83 (-5.29 to -1.49)	-	-
	33%	0.49	-6.58 (-7.78 to -1.66)	0.47	-5.28 (-8.48 to -1.57)
	66%	0.35	-4.06 (-6.85 to -0.06)	0.41	-4.41 (-8.65 to -0.37)
	100%	-	-	0.41	-5.14 (-8.78 to -0.60)
ESC	Baseline	0.33	2.45 (-3.61 to -0.58)	-	-
	33%	0.43	-3.70 (-6.23 to -1.77)	0.49	-4.41 (-7.46 to -1.12)
	66%	0.30	-3.30 (-5.70 to -0.21)	0.47	2.19 (-8.62 to –0.87)
	100%	-	-	0.43	-2.14 (-10.61 to –0.46)
RF	Baseline	-	-	-	-
	33%	0.32	-2.95 (-4.30 to -0.25)	0.39	-1.45 (-3.31 to –0.09)
	66%	0.40	-2.31 (-4.18 to -0.70)	0.37	-1.24 (-4.5 to -0.12)
	100%	-	-	-	-

#### Power spectrum density of sEMG signal during ISWT

**Fig 3:**
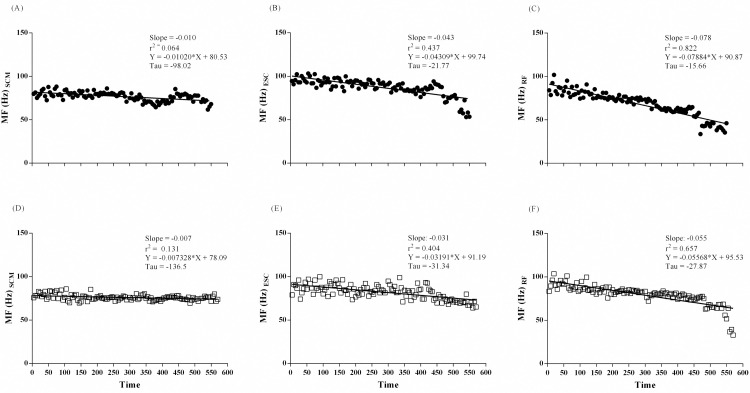
sEMG frequency median of SCM (A and D), ESC (B and E), and RF (C and F) muscles on ISWT, in Asthma-group (close circles) on upper panel and control group (open square) on the lower panel.

**Fig 4:**
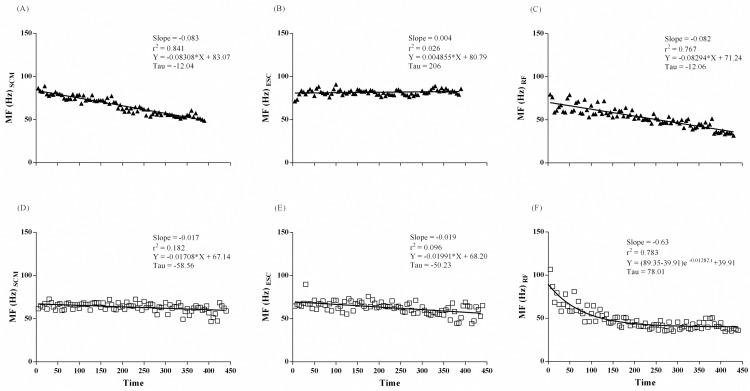
sEMG frequency median of SCM (A and D), ESC (B and E), and RF (C and F) muscles on ISWT, in COPD-group (close triangles) on upper panel and control group (open square) on the lower panel.

MF slopes decreased linearly in all muscles during ISWT. Moreover, intergroup differences were observed for ESC (p = 0.016) and RF (p < 0.0001) muscles with higher slopes in asthma group (Fig 3). MF decreased linearly in SCM and RF of COPD group. Slopes were different for SCM (p < 0.0001) in intergroup analysis with higher slopes in COPD group (Fig 4). Slopes of MF for RF between COPD subjects and controls were not calculated due to different decay patterns.

**Fig 5:**
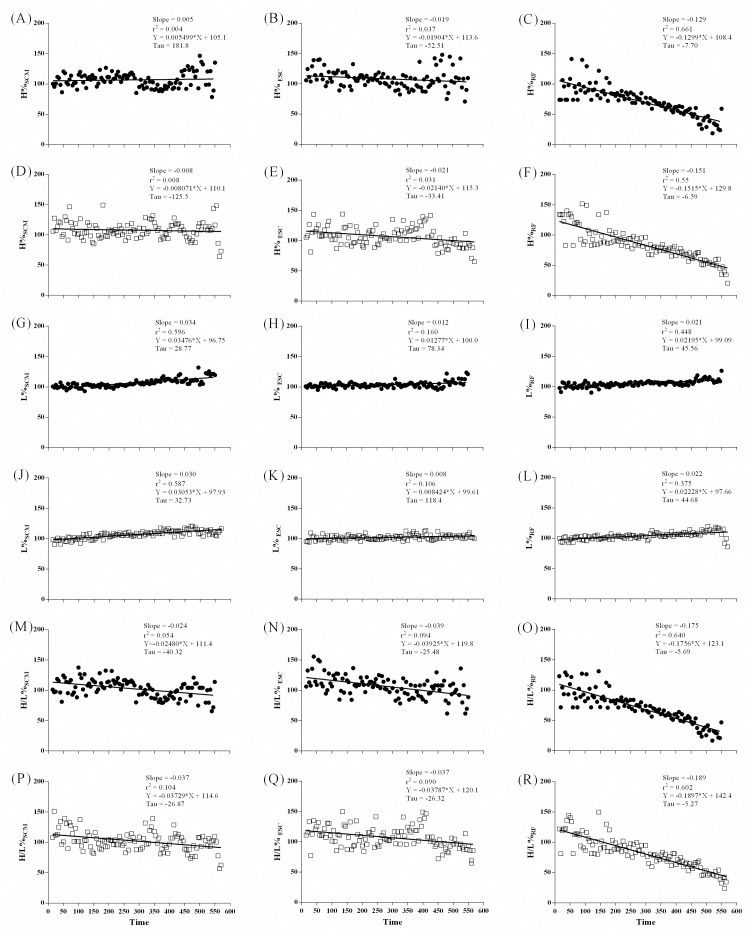
The high and low frequency bands, and high/low ratio of SCM (A, D, G, J, M and P), ESC (B, E, H, K, N and Q), and RF (C, F, I, O and R) muscles on ISWT, in Asthma-group (close circles) and control group (open square).

High-frequency band of ESC and RF decreased linearly in asthma group, whereas low-frequency band increased linearly in all muscles of both groups. H/L ratio decreased linearly in all assessed muscles of both groups. Slopes of power spectrum of assessed muscles were not different between groups (Fig 5).

**Fig 6:**
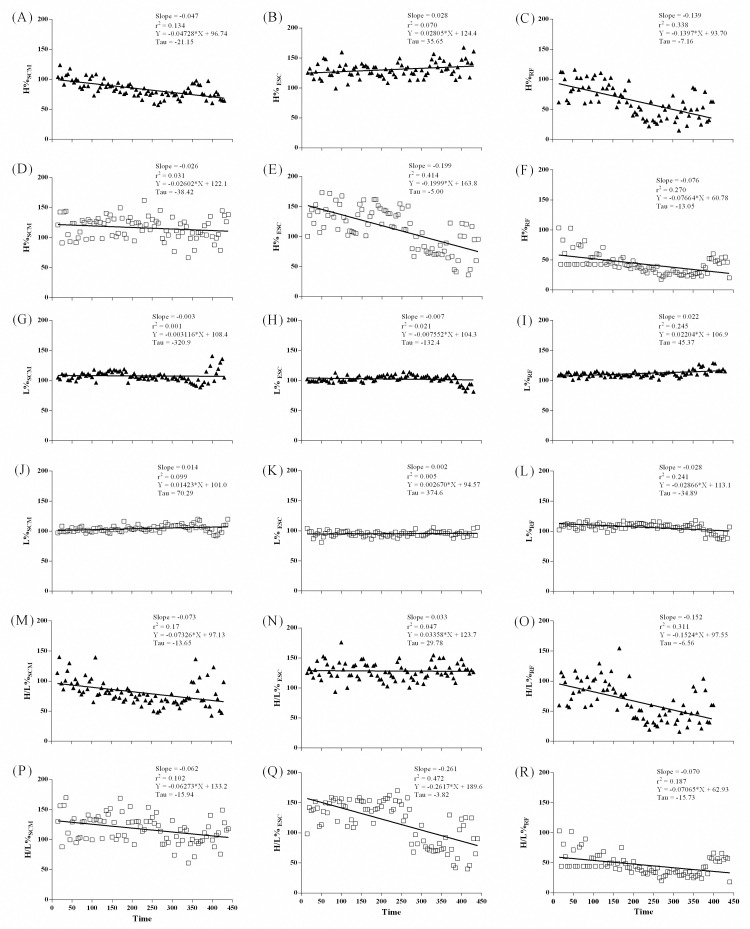
The high and low frequency bands, and high/low ratio of SCM (A, D, G, J, M and P), ESC (B, E, H, K, N and Q), and RF (C, F, I, O and R) muscles on ISWT moments, in COPD group (close triangles) and control group (open square).

High-frequency band decreased linearly in SCM and RF muscles of COPD group. Intergroup differences were observed in slopes of high-frequency bands of ESC (p <0.0001) and RF (p = 0.014). Low-frequency band of RF increased linearly in COPD group, and slopes were significantly greater in COPD group (p < 0.0001). H/L ratio of SCM and RF muscles decreased linearly in COPD group. COPD group exhibited greater slope values of H/L ratio in ESC (p < 0.0001) and RF (p = 0.002) compared with controls (Fig 6).

## Discussion

The present study aimed to assess surface electrical activity and fatigue development of respiratory (sternocleidomastoid and scalene) and locomotor (rectus femoris) muscles using sEMG during ISWT in individuals with obstructive respiratory diseases *versus* healthy individuals. Main findings of the present study were that 1) individuals with COPD and asthma presented greater sEMG amplitude of respiratory peripheral muscles during ISWT than healthy individuals; 2) individuals with asthma presented onset of fatigue development according to decreased MF of ESC and RF muscles; 3) fatigue development of SCM and RF in individuals with COPD were reflected by MF decrease and power spectrum distribution, respectively, compared with healthy individuals.

sEMG amplitude of respiratory muscles increased throughout ISWT and was higher in individuals with obstructive respiratory disease than healthy controls. Individuals with asthma have persistent inspiratory muscle activity [42]. In individuals with COPD, greater activation of ESC and intercostal muscles during maximum incremental exercise were associated with increased dyspnea [5]. Such findings corroborate those found in our study. Work of breathing increases during exercise due to functional respiratory muscle weakness (i.e., result of muscle shortening and abnormal geometry) in subjects with asthma and COPD [4, 43]; thus, increasing neural respiratory drive to diaphragm and pulmonary rib cage muscles (i.e., parasternal, intercostal, SCM, and ESC) [3, 44]. sEMG can accurately reflect neural respiratory drive to inspiratory muscles during exercise [5]. We believe that increased sEMG amplitude of respiratory muscles in individuals with obstructive respiratory disease reflects loads imposed on respiratory muscles triggered by mechanical abnormalities (dynamic hyperinflation, increased minute ventilation and respiratory rate, and intrinsic positive end-expiratory pressure) [6].

Activation of respiratory muscles was coordinated with RF in all individuals. Respiratory diseases impose a risk for locomotor muscle dysfunction [7, 12] due to physical inactivity, disuse, and chronic exposure to systemic corticosteroids. In the present study, individuals with chronic respiratory diseases were using medication to control the disease. Dysfunction of locomotor muscles includes changes in fiber distribution (from type I to type II), muscle atrophy and mitochondrial dysfunction, and reduced muscle capillarity and oxidative capacity [6, 7, 9]. Increased electrical activation corroborates the notion that atrophic muscles require increased activation for achieving a given strength or task than trained muscles [45]. Thus, we believe that greater activation of RF in this population is attributed to locomotor muscle deconditioning since these individuals are exposed to structural, metabolic, and energetic changes, mainly in the quadriceps femoris muscle.

MF of ESC and SCM reduced linearly during ISWT in asthma group and COPD groups, respectively, indicating fatigue development [36]. Despite the obstructive pattern, both diseases exhibited particularities that could explain these findings. Individuals with asthma were younger, presented lower obstruction levels (FEV_1_ = 77.20%) than those with COPD, and activated ESC during exercise. Although greater activation of SCM was also observed in individuals with asthma, this muscle may have not developed fatigue during ISWT. On the other hand, higher obstruction levels in COPD may have altered diaphragm geometry and reduced capacity of inspiratory muscles to generate negative intrathoracic pressure [46]. Consequently, greater rib cage muscle recruitment (including SCM) is needed to increase ventilation during exercise.

Moreover, SCM is composed of a larger proportion of type II fibers (65%) [47] than ESC (39%) [48]. Therefore, higher concentrations of metabolites (i.e., H+ ions and lactic acid) are produced, reducing intramuscular pH and muscle fiber conduction velocity, and shifting power spectrum to lower frequencies [21]. In this context, we suggest that fatigue development of SCM was higher in individuals with COPD, whereas fatigue development of ESC was more pronounced in asthmatic individuals.

The MF of RF of individuals with asthma and COPD also declined during the test. Although power spectrum and H/L ratio were similar between asthma and control groups, the decline in slope of MF was significantly higher in the former, which presented worse ISWT performance. H/L ratio and high- and low-frequency bands were different between COPD and control groups. Based on previous studies that observed a decline in MF of RF during dynamic contractions associated with power spectrum changes [49], we believe that declined MF found in our study was related to enhanced recruitment of slow-firing, fatigue-resistant motor units and/or reduced recruitment of rapid-firing, fatigable motor units. Therefore, RF developed fatigue during ISWT in both groups, but mainly in the COPD group, which experienced fatigue at the end of the test.

Susceptibility to lower limb muscle fatigue may be also related to blood flow distribution to respiratory muscles during exercise. Because of muscle metaboreflex activation, respiratory muscles initiate a blood flow "stealing effect" that compromises oxygen support and removal of metabolic by-products to activated locomotor muscles [16, 43, 50]. Based on recent evidence, the changes in power spectrum density of RF in individuals with obstructive respiratory disease may have been indirectly influenced by such events.

Our findings suggest that decreased MF and changes in power spectrum density of respiratory and locomotor muscles may be attributed to fatigue development during ISWT, especially in COPD individuals. In asthmatics, isolated analysis of MF decay in RF muscle provides incomplete information about changes in power spectrum density during fatigue development. Changes in frequency bands are attributed to underlying physiological mechanisms, but this information cannot be revealed if only MF is considered. To our knowledge, literature lacks simultaneous sEMG evaluation of respiratory and locomotor muscles during field walking tests in individuals with obstructive respiratory diseases. Our study helps to fill this gap in the literature since sEMG provides a non-invasive alternative for assessing the behavior of respiratory and locomotor muscles during dynamic exercise. Furthermore, sEMG data can assist in interpreting changes in activation and fatigue development of respiratory and locomotor muscles during rehabilitation.

This study has limitations that should be considered. We are aware that SCM and ESC might also have been activated during neck rotation when changing directions in the ISWT. Future research must include diaphragm, rib cage, and abdominal muscles [43] to enhance comprehension of respiratory muscle action during field walking tests. Besides, sEMG alone is insufficient to confirm muscle metaboreflex. Therefore, future researchers using sEMG combined with advanced resources to assess blood flow and respiratory and locomotor oxygenation (e.g., near-infrared spectroscopy—NIRS) are needed.

## Conclusion

Individuals with obstructive respiratory disease exhibit increased electrical activity of respiratory and locomotor muscles during ISWT compared with healthy individuals. Fatigue development in these muscles is also related to low performance in the ISWT.

## Supporting information

S1 File (RAR)
